# Surface and Interface Engineering for Organic Device Applications

**DOI:** 10.3390/ma14164647

**Published:** 2021-08-18

**Authors:** Ju-Hyung Kim

**Affiliations:** Department of Chemical Engineering and Department of Energy Systems Research, Ajou University, Suwon 16499, Korea; juhyungkim@ajou.ac.kr

In last few decades, organic materials (or carbon-based materials in a broad sense) including polymers have received much attention for their potential applications in electronics, because they have outstanding advantages such as high processibility, mechanical flexibility, and low weight. Extensive research efforts have thus been devoted to the development and advancement of organic materials for various applications, covering a wide range from molecular design to device-fabrication methods. In addition, it has been recognized that surfaces and interfaces play a crucial role in the operation and performance of the devices. For instance, various interactions at organic–metal interfaces are of great importance in organic epitaxy, and also have a strong correlation with intermolecular structures and their electronic properties.

In this context, the main focus of this Special Issue was collecting scientific contributions addressing surface and interface engineering with organic materials, and related applications. The diversity of contributions presented in this Special Issue exhibits the potential of organic materials in a variety of applications that are not limited to the fabrication of organic devices. This Special Issue contains eight featured original research papers as regards nanoarchitecture based on organic elements [1], physical behaviors of organic-based composite materials [2], surface exfoliation and device fabrication using functional polymers [3,4], performance enhancements of organic-based electronic devices [5,6], conductivity analysis of conducting polymers [7], and measurement system applicable to analysis of surface and interface engineering [8].

In the contributed article entitled “Investigation on the Adsorption-Interaction Mechanism of Pb(II) at Surface of Silk Fibroin Protein-Derived Hybrid Nanoflower Adsorbent” [1], Xiang Li et al. demonstrated the preparation of protein-derived hybrid nanoflowers via self-assembly, and investigated their adsorption behaviors and partialized functions of organic and inorganic elements upon adsorption. The prepared nanoflowers exhibited excellent efficiency for Pb(II) removal, which was evaluated by thermodynamic and adsorption kinetics investigation in this study.

Byung Soo Hwang et al. provided an outlook for the development of stimuli-responsive functional materials through their study of gelation behaviors of hydrogels in the contributed article entitled “Thermogelling Behaviors of Aqueous Poly(N-Isopropylacrylamide-co-2-Hydroxyethyl Methacrylate) Microgel–Silica Nano-particle Composite Dispersions” [2]. In this study, an in-depth investigation of the unique thermogelling behaviors of poly(N-isopropylacrylamide)-based microgels through poly(N-isopropylacrylamide-co-2-hydroxyethyl methacrylate) microgel (p(NiPAm-co-HEMA))-silica nanoparticle composite was performed with different molar ratios and various concentrations.

Peng Xiao et al. presented a facile method for the fabrication of liquid metal electrodes using polydimethylsiloxane, and also demonstrated solar-blind photodetection via surface exfoliation in a series of contributed articles entitled “Fabrication of a Flexible Photodetector Based on a Liquid Eutectic Gallium Indium” [3], and “Flexible and Stretchable Liquid Metal Electrodes Working at Sub-Zero Temperature and Their Applications” [4]. Two types of gallium-based liquid metal, eutectic gallium indium and galinstan, were employed for their works, and comparative studies according to material properties are described in the second article.

In the contributed article entitled “Crack-Assisted Charge Injection into Solvent-Free Liquid Organic Semiconductors via Local Electric Field Enhancement” [5], Ju-Hyung Kim et al. addressed surface engineering of metal electrodes for local electric field enhancements in organic devices. Efficient charge injection into solvent-free liquid organic semiconductors was demonstrated via intentionally cracked metal structures. It was also found that the cracked structures significantly increased the current density, which was strongly supported by a field intensity calculation.

In the contributed article entitled “Cesium Doping for Performance Improvement of Lead(II)-acetate-Based Perovskite Solar Cells” [6], Min-Seok Han et al. presented the preparation of organolead trihalide perovskite materials using lead(II)-acetate as a lead source for which inconvenient antisolvent treatment was not necessary. The effect of cesium doping on the performance of lead(II)-acetate-based solar cells was investigated, and a power conversion efficiency of 18.02% was eventually achieved in this work.

Hyeok Jo Jeong et al. reported the sigmoidal concentration dependence of electrical conductivity of poly(3,4-ethylenedioxythiophene):poly(styrene sulfonate) processed with linear glycol-based additives in the contributed article entitled “Sigmoidal Dependence of Electrical Conductivity of Thin PEDOT:PSS Films on Concentration of Linear Glycols as a Processing Additive” [7]. It was found that repeated ethylene oxides with hydroxyl groups were effective in enhancing the electrical conductivity of poly(3,4-ethylenedioxythiophene):poly(styrene sulfonate) in this work.

In the contributed article entitled “The Methods and Experiments of Shape Measurement for Off-Axis Conic Aspheric Surface” [8], Shijie Li et al. presented three methods (i.e., auto-collimation, single computer-generated hologram, and hybrid compensation) to test the shape accuracy of the off-axis conic aspheric surface with high precision. It was demonstrated that their experimental results involved in peak-to-valley, root-mean-square, and shape distribution were consistent in the three different methods.

In conclusion, the contributed articles in this Special Issue demonstrate relevant progress and the potential of organic materials in a variety of applications. I wish to thank and acknowledge all the authors for their priceless contributions and the editorial members of *Materials*. I am personally grateful to Ms. Freda Zhang, Managing Editor of this Special Issue.

## Data Availability

Not applicable.
